# Regulation of term and preterm labor: genetics and epigenetics

**DOI:** 10.3389/fgene.2025.1594030

**Published:** 2025-06-18

**Authors:** Kaiyi Sun, Ning Wang, Yuanyuan Liu, Lu Gao

**Affiliations:** ^1^ Department of Physiology, College of Basic Medical Sciences, Naval Medical University, Shanghai, China; ^2^ Shanghai Key Laboratory for Assisted Reproduction and Reproductive Genetics, Shanghai Jiaotong University, Shanghai, China

**Keywords:** labor, preterm birth, genetics, epigenetics, term labor

## 1 Introduction

The occurrence of labor is a highly coordinated process involving multiple physiological systems. Abnormal parturition can result in adverse pregnancy outcomes, including preterm birth (PTB), dystocia, and post-term birth, which directly impact the health and survival of both the mother and the fetus (Chawanpaiboon et al., 2019). Among these, PTB is one of the most prevalent pregnancy complications, affecting approximately 5%–18% of pregnant women worldwide and significantly increasing the risk of neonatal morbidity and mortality (Romero et al., 2014).

Non-genetic risk factors for PTB include inflammation, immune-related pathways and abnormalities in decidual structure or function. However, advancing research has identified genetic and epigenetic factors that also modulate the functions of various tissues within maternal and fetal systems. Moreover, interactions between genetic/epigenetic factors and environmental influences, such as obesity and smoking, have been implicated in PTB, underscoring their significance in parturition (Li et al., 2022). Therefore, systematically summarizing existing research and exploring the mechanisms underlying the initiation of labor and PTB will provide an effective foundation for PTB prevention and treatment.

## 2 Maternal genetic factors contributing to labor

Genome-wide association studies (GWAS), along with expanded whole-exome sequencing (WES) and whole-genome sequencing (WGS), have provided powerful tools for identifying genetic pathways related to birth timing and PTB. A Large-scale GWAS has identified several maternal genetic variants loci associated with gestational duration and PTB risk, including EBF1, EEFSEC, AGTR2, WNT4, ADCY5, and RAP2C (Zhang et al., 2017). Some of these loci have also been validated in cohort studies of spontaneous preterm birth (sPTB) (Ehret et al., 2011; Jain et al., 2022). However, GWAS research on PTB remains limited, primarily due to the small scale or restrictions of single ethnic groups or geographical regions, leading to a lack of replication, particularly in high-risk sPTB populations.

Genetically engineered mouse models offer a valuable approach for elucidating the mechanisms of genetic disorders. In CXCR3-deficient mice, the sPTB-associated cytokines were not increased acutely in amniotic fluid following the injection of lipopolysaccharide (LPS) at a preterm birth-inducing dose, suggesting that CXCR3 contributes to sPTB (Karjalainen et al., 2015). Hirota et al. conditionally knocked out the *Trp53* gene in mouse uteri using Pgr-cre and found that Trp53-deficient mice exhibited a significant predisposition to PTB, with approximately 50% delivering as early as embryonic day 16.5 (Hirota et al., 2010). Since *Trp53* (*TP53* in human) is a classical aging-related gene, this finding also provides insight into the increased PTB risk observed in older pregnant women (Supplementary Table S1).

## 3 Fetal genetic factors contributing to labor

Although the maternal genome exerts a predominant influence on gestational duration and PTB occurrence, emerging evidence suggests that fetal genetic factors also play a critical role. A previous study from our group demonstrated that wild-type female mice mated with male mice deficient in steroid receptor coactivators 1 and 2 (SRC-1 and SRC-2) exhibited significantly reduced secretion of surfactant protein A (SP-A) and platelet-activating factor (PAF) in fetal lung, leading to a severe delay in parturition of approximately 38 h by attenuating the inflammatory pathway in the myometrium (Gao et al., 2015), which highlighted the significant roles of fetus-derived factors in triggering the initiation of labor in murine model. Interestingly, a high-resolution metabolic profile covering the whole gestational period has revealed the metabolic characteristics of pregnant women and facilitated the construction of a metabolic clock for predicting gestational age. This study found that the plasma level of PAF increases according to human gestational age (Liang et al., 2020), suggesting that the dynamic changes of PAF may be closely associated with parturition in human as well. Moreover, another study demonstrated that PAF in fetal cord blood from term not in labor women was significantly higher than that from preterm not in labor women, which was further increased in term in labor women. Of more interest, it was found that concentrations of PAF from umbilical venous blood were significantly lower than umbilical arterial blood from the same patients, further confirming our previous findings in mouse model that the increased PAF are contributed primarily by the fetus, other than from the maternal side. Furthermore, PAF can induce DNA methylation in the promoter region of the progesterone receptor (PR-A) in human placental tissue, leading to the suppression of PR-A transcription, which may represent a non-classical functional progesterone withdrawal mechanism comparing to that in myometrium (Palomares et al., 2021). These follow-up researches, combined with our original findings, strongly suggest that fetus-derived PAF plays critical roles in triggering parturition in both mouse and human, although the underlying mechanisms may differ between mouse and human.

In a study of Finnish families with multiple cases of sPTB identified a genetic linkage between sPTB and specific genes encoding the insulin-like growth factor 1 receptor (IGF1R) and androgen receptor (AR) in fetal genome. These findings were replicated in case-control studies of another Finnish sPTB cohort (Haataja et al., 2011; Karjalainen et al., 2012; Aguirre et al., 2016). Additionally, a large-scale GWAS conducted on infants born preterm, identified a fetal locus on chromosome 2q13 as a crucial region associated with labor onset (Liu et al., 2019) (Supplementary Table S1). These findings suggest that fetal genetic factors also play a critical role in regulating parturition. Future genetic studies on PTB should comprehensively consider the contributions of both maternal and fetal genomes.

## 4 Epigenetic regulation of term or preterm labor timing

### 4.1 MicroRNA

During late pregnancy or near delivery, the upregulation of oestradiol-17β (E_2_) suppresses the expression of zinc finger E-box binding homeobox 1 and 2 (ZEB1 and ZEB2). This suppression results in an increased expression of the miR-200 family, which in turn inhibits the activation of signal transducer and activator of transcription 5B (STAT5B). Subsequently, this process enhances the expression of progesterone-metabolizing enzyme 20α-hydroxysteroid dehydrogenase (20α-HSD), reducing the local progesterone levels and ultimately triggering labor (Williams et al., 2012). Simultaneously, the suppression of ZEB1 decreases the expression of miR-199a-3p and miR-214, in turn leading to the increase in expression of cyclooxygenase-2 (COX-2), which promotes myometrial contraction and preterm labor (Renthal et al., 2010). Towards the end of labor, the enhancement of estradiol-17β/ERα signaling pathway in myometrium suppresses the expression of miR-181a. The reduction in miR-181a levels, in turn, upregulates the expression of estrogen receptors-α (ERα), tumor necrosis factor α (TNF-α), and other pro-inflammatory cytokines, which may contribute to the initiation of preterm birth (Gao et al., 2016).

A range of microRNAs exhibiting significant differential expression have been identified in the serum of pregnant women experiencing PTB, including miR-200a, miR-4695-5p, miR-665, and miR-887 (Elovitz et al., 2015). However, the expression patterns of some miRNAs in the serum of PTB cases do not align with phenotypes observed in animal models. For example, in murine models, the upregulation of miR-200a enhances local progesterone metabolism and suppresses ZEB1/2 expression, thereby increasing myometrial contractility and promoting the transcription of genes involved in uterine contraction (Renthal et al., 2010; Williams et al., 2012; Renthal et al., 2013). In contrast, a downregulation of miR-200a has been observed in the serum of women with PTB, albeit to a lesser extent (Elovitz et al., 2015). Due to the complexity of gene mutations and interspecies homology differences, mouse models may not always accurately mimic human phenotypes (Supplementary Table S1). Therefore, while numerous miRNAs have been implicated in the regulation of labor onset and PTB, it is essential to consider the conservation and divergence of regulatory mechanisms across different tissues, organs, and species when extrapolating findings from model organisms to human physiology.

### 4.2 LncRNA

Long non-coding RNAs (lncRNAs) play a crucial role in preterm premature rupture of membranes (PPROM) by regulating the mRNA levels in pathways associated with extracellular matrix (ECM) remodeling, apoptosis, actin cytoskeleton organization, and smooth muscle contraction (Luo et al., 2015). The expression of lncRNA 504601 and CR602937 is significantly upregulated in patients with PPROM, leading to a downregulation of collagen family members COL1A1 and COL18A1. Since these collagens are directly involved in ECM formation and remodeling, their degradation is a major factor contributing to fetal membrane reconstruction and may underlie the pathogenesis of PPROM (Zhao et al., 2017).

Zhou et al. identified five circadian rhythm-associated lncRNAs (INC00893, LINC00265, LINC01089, LINC00482, and LINC00649) that were significantly downregulated in the placenta of sPTB cases by analyzing two independently published RNA datasets (GSE73712 and GSE174415) (Zhou et al., 2022). These findings suggest a correlation between changes in lncRNA expression levels and the occurrence of preterm birth (Supplementary Table S1). However, further research is required to elucidate the regulatory mechanisms underlying lncRNA expression, which may provide reliable evidence for developing specific therapeutic targets for preterm birth prevention and management.

### 4.3 DNA methylation

In the amnion of preterm patients, the promoter region of the oxytocin receptor (OXTR), a classical contraction-associated protein, exhibits increased DNA methylation levels (Kim et al., 2013). Given that DNA methylation generally plays a role in gene silencing, although the expression of OXTR significantly increased near labor, the DNA methylation observed in *OXTR* gene may not correlate with OXTR expression. Moreover, the CpG island of solute carrier family 30 member 3 (SLC30A3) also exhibits hypermethylation in the amnion of preterm patients, leading to reduced expression of SLC30A3 (Kim et al., 2013). As a zinc transporter, the dysregulated expression of SLC30A3 may disrupt nutritional homeostasis during pregnancy, ultimately leading to PTB. In amnion fibroblasts from patients with PPROM, a decrease of methylation levels in the *MMP1* gene promoter region has been observed, resulting in a marked increase in transcription of *MMP1* gene. MMP1 facilitates the degradation of fibrillar collagen, which is associated with the breakdown of the extracellular matrix of the fetal membranes (Wang et al., 2008). The differential regulatory patterns of DNA methylation across various tissues during pregnancy provide epigenetic insights into the timing of labor and the occurrence of preterm birth (Supplementary Table S1).

### 4.4 Histone modification

During labor, activating histone modifications such as H3K4me3 (trimethylated histone H3 lysine 4) and H3/H4 acetylation occur in the PR-A promoter region of myometrium, leading to an upregulation of PR-A mRNA levels. This results in an increased PR-A/PR-B ratio in the myometrium, as PR-B mediates the classical actions of progesterone, including the maintenance of myometrial quiescence, while PR-A acts as an inhibitor of progesterone action mediated by PR-B and the functional withdrawal of progesterone is mediated by the enhanced expression ratio of PR-A/PR-B (Pieber et al., 2001), which characterizes myometrial activation and the initiation of labor (Chai et al., 2012). The dissociation of JARID1A from the PR-A promoter facilitates H3K4 trimethylation and histone acetylation, thereby enhancing PR-A expression in the myometrium and further elevating the PR-A/PR-B ratio. This mechanistic process explains the increased H3K4me3 levels and PR-A expression observed in both term and preterm labor (Chai et al., 2014). Another study reported that the expression of histone deacetylase HDAC1 gradually decreases throughout pregnancy and HDAC1 can bind to the PR-A promoter region, thereby upregulating PR-A expression (Ke et al., 2016). These findings suggest that histone methylation or acetylation may contribute to progesterone functional withdrawal, a critical process in human parturition.

For years, research on parturition timing has primarily focused on proximal triggers of labor onset in late pregnancy. Recent studies have revealed that histone demethylase KDM6B regulates H3K27me3 levels in uterine fibroblasts during early pregnancy (GD 0.5–GD 5.5) in mice. In KDM6B-deficient mice, excessive accumulation of H3K27me3 at multiple genomic loci led to transcriptional alterations in late pregnancy, including dysregulation of Ptgs1, COX-1, PGF2α and Akr1c18, as well as delayed serum P4 decline, ultimately resulting in 1–3 days delay in parturition (McIntyre et al., 2025). This interesting study sheds new light that epigenetic regulation during early pregnancy may provide reliable evidence for the early prediction of parturition timing or PTB risk (Supplementary Table S1).

### 4.5 The association between epigenetic alterations in maternal diseases and preterm birth

Maternal disorders such as pre-eclampsia (PE), gestational diabetes mellitus (GDM), and intrauterine infections are the leading causes of iatrogenic PTB, which can independently alter widespread epigenomics, highlighting the complex interplay between maternal health and epigenomic regulation during pregnancy (Vogel et al., 2018).

PE is the major cause of maternal mortality in developed countries and contributes significantly to iatrogenic PTB and low birthweight infants, affecting approximately 3%–5% of pregnant women (Dimitriadis et al., 2023). Studies have shown that DNA methylation in the promoter region of TIMP3 is significantly reduced in the placentas of women with PE (Cruz et al., 2022). However, some studies observed higher whole DNA methylation in placentas from women with PE (both term and preterm labor) when compared with normotensive women, whereas no association of placental methylation patterns with birth outcome (Kulkarni et al., 2011). Despite conflicting findings regarding whole DNA methylation levels in the placentas of PE largely attributable to methodological differences, no definitive association of placental DNA methylation patterns with birth outcomes has been established. (Kulkarni et al., 2011). Pineles et al. were the first to report seven differentially expressed miRNAs in placentas from women with PE who delivered either normal infants or small-for-gestational-age (SGA) infants. Notably, miR-210 and miR-182 were significantly upregulated in women with PE and PE + SGA compared to those with sPTB (Pineles et al., 2007). MiR-374a-5p and miR-191-5p have been implicated in both PE and PTB, suggesting a possible molecular connection between the two conditions (Subramanian et al., 2023). In addition, histone modifications such as H3K4me3, H3K9ac, and H3K27me3 were significantly reduced in the placentas of women with PE, independent of fetal sex (Meister et al., 2021; Deng et al., 2023). However, the epigenetic mechanisms linking PE to PTB remain underexplored, underscoring the need for more studies to provide definitive and clinically relevant epigenomic evidence (Supplementary Table S1).

Gestational diabetes mellitus (GDM) is another common pregnancy complication strongly associated with PTB and other adverse outcomes. Epigenomic alterations in various tissues and cells have been shown to play pivotal roles in GDM pathogenesis and may influence long-term health in offspring (Abu Samra et al., 2022). A study focusing on African American women found that methylation of the CYTIP and LINC00114 gene promoters in whole blood was associated with PTB in the context of GDM. These modifications may promote inflammatory signaling and premature uterine contractility, thus triggering preterm labor (Hong et al., 2018). Furthermore, analyses of umbilical cord blood and tissue from preterm infants suggest a prominent role for DNA methylation in immune and inflammatory pathways (Wu et al., 2019). Epigenetically modified genetic material in GDM pregnancies may contribute to PTB by enhancing inflammatory responses, supporting the concept that maternal metabolic and immune status directly influences birth timing through epigenetic regulation (Supplementary Table S1).

## 5 Genetic-based screening strategies for prediction of preterm birth

Given the association between maternal genetic factors and preterm birth, as well as the exclusive maternal inheritance of mitochondrial DNA (mtDNA) mutations, which have been linked to various hereditary diseases, Yang et al. established an automated pipeline for detecting mitochondrial DNA (mtDNA) mutations using low-coverage whole-genome sequencing (lcWGS) data. Applying lcWGS to a cohort of 929 PTB samples (21–30 weeks) from diverse ethnic backgrounds, they identified mtDNA variants potentially contributing to PTB, including known pathogenic variants in 8 cases and rare variants in 47 cases (Yang et al., 2021). This study provides an alternative genetic screening approach for investigating parturition and PTB.

Clinical studies have documented that cell-free fetal DNA (cfDNA) levels increase with gestational age and peak at the end of pregnancy (Phillippe, 2014). Additionally, research has shown that the plasma cfDNA concentration in women undergone preterm labor is twice that of those undergone term (Leung et al., 1998). Furthermore, studies also have demonstrated that patients with abnormally elevated cfDNA levels in mid-pregnancy face a significantly increased risk of sPTB in late pregnancy (Jakobsen et al., 2012). These findings indicate cfDNA can serve as a potential biomarker for labor initiation. The underlying mechanism likely involves the activation of pattern recognition receptors, such as Toll-like receptor 9 (TLR9) or other DNA-sensing pathways, including the STING signaling pathway, by freely circulating hypomethylated DNA, subsequently triggering downstream cascades leading to labor onset (Bauer et al., 2006; Fűri et al., 2015; van Boeckel et al., 2018). As a result, the application of cfDNA provides a non-invasive genetic screening approach for PTB prediction, substantially reducing the need for invasive diagnostic procedures such as amniocentesis while offering objective evidence for early medical intervention, thereby contributing to improved PTB prevention strategies (Tarca et al., 2021).

In recent years, non-invasive prenatal testing has expanded to measurements of circulating RNA in whole blood or plasma cell-free RNA (cfRNA) (Moufarrej et al., 2020). Large-cohort studies have successfully identified women at risk of preterm birth over 2 months in advance by measuring cfRNA levels, demonstrating the predictive potential of cfRNA for labor timing. However, existing studies have primarily focused on spontaneous preterm birth within specific ethnic groups, necessitating larger-scale validation and longitudinal studies across diverse populations to enhance the clinical applicability of cfRNA (Ngo et al., 2018). In another prospective study, maternal blood samples were collected during mid-pregnancy for cfRNA analysis, leading to the establishment of a cfRNA transcriptomic profile. This study identified multiple transcripts and regulatory pathways associated with sPTB, including extracellular matrix organization and degradation pathways and the insulin-like growth factor signaling pathway (Camunas-Soler et al., 2022). These findings provide an extensive time window for clinical intervention, as early pregnancy plasma cfRNA measurements could offer predictive insights into late-gestation clinical outcomes. Specifically, our recent study by analyzing public cfRNA database revealed that the cfRNA levels of TNFSF4 in maternal peripheral blood are elevated during preterm labor compared to term labor, suggesting the TNFSF4 could serve as a novel cfRNA biomarker for non-invasive prenatal diagnosis of preterm birth (Wang et al., 2023). However, its specificity and sensitivity require further validation with additional clinical data.

## 6 Conclusion

The prevention and management of PTB remain global challenges. Investigating genetic and epigenetic alterations associated with parturition initiation and PTB will enhance our understanding of the molecular mechanisms underlying sPTB and its long-term health consequences for offspring. It is increasingly evident that both genomic and epigenomic changes play crucial roles in the pathogenesis of PTB. Although advances in high-throughput technologies have generated vast amounts of data and identified numerous potential genetic and epigenetic loci for diagnosis and prevention of PTB, the lack of effective integration of global genomic data due to the volume and complexity may limit their translation into clinical practice. Therefore, leveraging artificial intelligence to integrate and model these large-scale genomic datasets holds great promise for the development of precise screening, diagnostic, and therapeutic strategies for PTB. In addition, despite growing evidence supporting the role of epigenetic modifications in the etiology of PTB, the dynamic nature of the epigenome during the prolonged and complex course of pregnancy and the high cost of testing have hindered the widespread clinical adoption of epigenomic analysis. Carter et al. have proposed the development of a commercially available, affordable, and standardized chromatin DNA reference sample to serve as a benchmark or threshold, which would enhance the standardization and reliability of epigenomic testing and facilitate its broader clinical implementation (Carter et al., 2023). In the future, the establishment of genetic and epigenetic monitoring studies spanning the entire gestational period could facilitate the identification of valuable genetic and epigenetic biomarkers, ultimately improving PTB prediction and prevention efforts.
